# A qualitative description of the daily living functioning of people diagnosed with borderline personality disorder from consumer, carer and clinician perspectives

**DOI:** 10.1111/1440-1630.70127

**Published:** 2026-09-28

**Authors:** Dillon Tepper, Ben Sellar, Sheryl Shipley, Rachel Smith, Carolyn M. Murray

**Affiliations:** ^1^ Adelaide University School of Allied Health and Human Performance Adelaide South Australia Australia

**Keywords:** activities of daily living, independent living, mental health recovery, psychiatric rehabilitation, qualitative research

## Abstract

**Introduction:**

Borderline personality disorder (BPD) is a severe and prevalent mental illness. Longitudinal research consistently demonstrates that people diagnosed with BPD experience persistent functional impairment despite current psychotherapeutic treatment. Functional impairment is typically defined in terms of performance in vocational and relational domains. However, functional recovery across multiple domains of functioning should also be considered, such as leisure, self‐care, health and household management. This study aimed to describe the daily living functioning of people diagnosed with BPD from multistakeholder perspectives.

**Methods:**

This qualitative descriptive study used semi‐structured interviews with 22 participants, including consumers (*n* = 4), carers (*n* = 5) and clinicians (*n* = 13). Data were analysed using reflexive thematic analysis.

**Consumer and community involvement:**

Consumer, carer and clinician consultants reviewed a draft of the interview guide and provided feedback on the terminology, question ordering and provision of practical examples. Their feedback was incorporated and amendments were made prior to data collection.

**Findings:**

Five themes were generated: (1) structured routines, (2) extremes of doing, (3) a lot of energy required to manage in daily life, (4) doing when disconnected from values and identity and (5) doing through others. People diagnosed with BPD experienced challenges establishing and maintaining structured routines, engaged in boom‐and‐bust activity patterns, expended considerable energy managing emotions and interpersonal interactions, struggled to identify values to derive meaning from daily activities and relied on others to initiate and complete daily activities.

**Conclusion:**

Functional recovery outcomes are important for people diagnosed with BPD. Occupational therapists are well positioned to support functional recovery for this population, which could involve exploring daily living functioning beyond a psychotherapeutic framework, including the structure of routines, sleep patterns, activity pacing, energy conservation, values identification, and the extent of carer involvement in daily activities.

## PLAIN LANGUAGE SUMMARY

Borderline personality disorder (BPD) is a serious mental health condition. The main treatment for BPD is talk therapy, but this does not always improve daily living. Research shows that many people diagnosed with BPD still struggle to get a job and be in a relationship despite talk therapy, which is what most research focuses on. We argue that there is more to daily life than working and being in a relationship. To get a fuller picture of what daily life looks like for people diagnosed with BPD, we interviewed people with a lived and living experience of a BPD diagnosis (consumers), carers and clinicians to get their perspectives on the topic. We looked closely at what participants said. We found that some people diagnosed with BPD might have problems maintaining a structured daily routine; overdo things when feeling good, then crash and do very little; find it exhausting to manage emotions and relationships (leaving not much energy for other daily activities); and struggle to identify what is important, making it hard to find meaning in daily activities; and some carers might be ‘doing for’ their person to start and finish daily activities. Occupational therapists could support people diagnosed with BPD by helping them with things like building structured routines, improving sleep, pacing everyday activities, managing energy, figuring out what is important, and working with carers when appropriate. A strength of this study is that we spoke with consumers, carers and clinicians. A limitation is that we interviewed more clinicians than consumers and carers. However, efforts were made to feature all voices when reporting the findings.

**Key Points for Occupational Therapy**

- Occupational therapists must explore the daily living functioning of people diagnosed with BPD beyond psychotherapeutic, vocational and relational frameworks.
- Effective rehabilitation could address balance and structure in routines, sleep patterns, activity pacing and values clarification.
- Appropriate carer involvement could support greater autonomy in daily activities.

## 1 INTRODUCTION

Borderline personality disorder (BPD) is a severe mental illness characterised by emotional dysregulation, interpersonal hypersensitivity, impaired mentalisation, early maladaptive schemas and identity diffusion (American Psychiatric Association, 2013; Leichsenring et al., 2024; World Health Organization, 2022). The estimated prevalence of BPD in the general population is between 0.7% and 2.7% yet, in psychiatric settings, accounts for up to 12% of outpatients and 22% of inpatients (Leichsenring et al., 2024). Suicide risk is markedly high for people diagnosed with BPD, with 80% experiencing ideation, 50% attempting and 6% dying by suicide (Lak et al., 2025).

Treatment for BPD is challenging. Medications do not alter the course or severity of BPD (Gartlehner et al., 2021; Stoffers‐Winterling, Ribeiro, et al., 2022). Clinical practice guidelines consistently recommend psychotherapy as the most viable treatment (Aslam et al., 2025; Louise et al., 2026). However, almost a third of people do not complete psychotherapy, and even for those who do, only half respond to treatment (Dixon & Linardon, 2020; Iliakis et al., 2021; Woodbridge et al., 2022). Psychotherapy can reduce symptom severity, self‐harm and suicidality, and some trials report improvements in ‘psychosocial functioning’ (Stoffers‐Winterling, Storebø, et al., 2022; Storebø et al., 2020). However, there is a persistent mismatch between symptomatic and functional recovery, with decades of prospective longitudinal research consistently showing that symptomatic remission is common and relatively stable, whereas social and vocational functioning is achieved by far fewer people and is less readily sustained (Álvarez‐Tomás et al., 2019; Choi‐Kain et al., 2020; Gunderson et al., 2011; Zanarini et al., 2010). The trial effects on ‘psychosocial functioning’ do not resolve this, as they are typically short‐term and often derived from the clinician‐rated Global Assessment of Functioning Scale, which conflates symptoms with functioning and does not capture the complexity of functioning across multiple domains of daily living (Desrosiers et al., 2020; Endicott et al., 1976).

In occupational therapy, functional impairment is addressed in any aspect of daily living. However, in the BPD literature, functional impairment is more narrowly defined, focussing on vocational performance and establishing and maintaining relationships (Culina et al., 2024; Dhar et al., 2023; Grenyer et al., 2022). Such a narrow definition of ‘work and love’ overshadows other domains of daily living such as physical activity, health‐care utilisation, leisure, creativity, household management, self‐care, sleep, life balance, parenting and financial management (Álvarez‐Tomás et al., 2024; Birken & Harper, 2017; Borovica et al., 2024; King et al., 2024; Larivière et al., 2016; Ludowyke et al., 2024; Petersen et al., 2025; Sansone & Wiederman, 2012; Steele et al., 2019; Wood et al., 2015). Rehabilitation goals and outcomes in these ‘other’ domains are also important.

Occupational therapists are well suited to support the functional recovery needs of people diagnosed with BPD across multiple domains of daily living (Weideman, 2025). However, as psychotherapeutic approaches currently dominate the evidence base, access to rehabilitation services to support daily living functioning might be limited (Choi‐Kain et al., 2017; Storebø et al., 2020). Moreover, many health professionals continue to harbour stigmatising attitudes and beliefs towards this population (Klein et al., 2022; Stiles et al., 2023). If clients were to meet access for a rehabilitation program, then the program would be service‐specific and lack rigorous evaluation (Tepper et al., 2025).

We argue that people diagnosed with BPD face persistent challenges in daily living functioning because of a lack of understanding about their rehabilitation needs. Qualitative inquiry is needed to gain a nuanced and comprehensive understanding of this. Such insights could support targeted, responsive rehabilitation programs and outcome measures that prioritise functional recovery. The aim of this study was to describe the daily living functioning of people diagnosed with BPD from multistakeholder perspectives. The research question was as follows: ‘How do consumers, carers and clinicians describe the daily living functioning of people diagnosed with BPD?’

## 2 METHODS

### 2.1 Study design

This study used a qualitative descriptive design underpinned by social constructionism (Burr & Dick, 2017; Sandelowski, 2000, 2010). The University of South Australia Human Research Ethics Committee approved this study (206296). Reporting followed the COREQ guidelines (Tong et al., 2007).

### 2.2 Positionality statement

The first author (D.T.) is a male occupational therapist with 6 years of clinical mental health experience. He is certified in dialectical behaviour therapy (DBT) and good psychiatric management (GPM) and has completed at least foundational training in mentalisation‐based treatment, schema therapy and transference‐focussed psychotherapy (Choi‐Kain et al., 2017). His clinical experience in supporting people diagnosed with BPD initiated this research. DT holds recovery‐oriented values and approached the data with an occupational therapy lens that focuses on the meaning of everyday activities. His occupational therapy and psychotherapy training predisposed him towards clinical framings of the findings. Thus, prior to analysis, DT reflexively examined these assumptions that would influence interpretation (Braun & Clarke, 2022). These assumptions were scrutinised through a multidisciplinary approach (S.S. and R.S.) and the qualitative occupational therapy perspectives of coauthors B.S. and C.M. The other researchers were a counselling psychologist (S.S.), who is psychoanalytically trained and an advanced practitioner in interpersonal psychotherapy; a nurse practitioner (R.S.), who is trained in DBT and GPM; and two occupational therapists (B.S. and C.M.), who have expertise in qualitative research.

### 2.3 Sampling and recruitment

Consumers were people with a lived and living experience of a BPD diagnosis. Carers provided care for a person diagnosed with BPD. The participant information sheets for the consumers and carers provided state and national crisis contacts and counselling services. Participant safety and well‐being were prioritised over interview completion; there were no known adverse events arising from interview participation. Clinicians were nurses, occupational therapists, psychiatrists, psychologists and social workers. Purposive, convenience and maximum variation sampling methods were used (Portney, 2020), aiming to recruit 30 participants: consumers (*n* = 10), carers (*n* = 10) and clinicians (*n* = 10). Within the clinician cohort, maximum variation sampling aimed for two of each discipline. Participants were recruited through social media and professional networks.

### 2.4 Inclusion criteria

Inclusion criteria for all stakeholder groups were 18 + years of age and living or working in Australia. Consumers had a self‐reported BPD diagnosis. Carers self‐reported providing care to a family member or friend diagnosed with BPD. Clinicians self‐reported that they had worked with at least one person diagnosed with BPD within the last 5 years.

### 2.5 Development of interview guide

The interview guide was developed with feedback from consumer, carer and clinician consultants known to the first author. The consumer consultant did not recommend any changes. The carer consultants recommended swapping the order of two interview questions, as well as providing more practical, activity‐based examples beneath some questions. The clinician consultant provided feedback on the terminology, phrasing and ordering of the questions. The consultants' feedback was incorporated and amendments were made. Table 1 shows the final consumer version of the interview questions. These questions were transposed into third person for the carers and clinicians.

**TABLE 1:** Interview questions (consumer version).

1. What does a typical day look like from when you wake up to when you go to sleep?
2. I want to understand what everyday life is like for people diagnosed with BPD. In your own words, please describe your life as you see it.
3. How do you decide to spend your time?
4. What daily activities are important to you?
5. How has your daily life changed over time? How did you adjust?
6. What moments in your daily life do you find meaningful?
7. What aspects of your daily life would you like to change?
8. What other aspects of daily living shape your experiences?
*The carer and clinician versions were transposed into third person*.

### 2.6 Data collection

All semi‐structured interviews were conducted by the first author (D.T.) via audio‐recorded Zoom or phone calls. Interviews were conducted between November 2024 and March 2025. All participants provided written informed consent and were eligible for payment.

### 2.7 Data analysis

Microsoft Word was used for data analysis, following Braun and Clark's (2022) six phases of reflexive thematic analysis: (1) The first author (D.T.) conducted and transcribed all interviews verbatim, reading transcripts multiple times. (2) Transcripts were coded line‐by‐line to identify discrete meaning units. A subset of four transcripts was coded independently by two other research team members (B.S. and C.M.): Any coding discrepancies were resolved through discussion and adjudicated by D.T. (3) Team meetings were held to cluster codes into categories, which D.T. summarised narratively. (4) A mind map was used to identify meaningful connections across categories to form candidate themes. (5) Candidate themes were summarised, named and defined. (6) The themes were then refined and finalised during write up, with checking undertaken to ensure quotes were included from all cohorts where possible. These phases were iterative, recursive and non‐linear (Braun & Clarke, 2022). There were regular team discussions throughout. D.T. maintained reflexive journals, which were shared with the research team (Braun & Clarke, 2022; Trainor & Bundon, 2021). Recruitment was ceased once there was sufficient data for thick description and to respond to the research question; data saturation was not sought because it is not a requirement of qualitative descriptive research (Stanley, 2023). A plain language summary of the final themes was shared with interested participants for member checking, inviting participants to provide feedback if desired. One carer responded, who confirmed that the findings were ‘… very informative and accurate. Now for the next step in how to assist those with BPD to enable them to live life and combat these difficulties …’.

## 3 FINDINGS

### 3.1 Participants

There were 22 participants (as shown in Table 2): four consumers (two female and two male); five carers (all female, who cared for two females and three males); and 13 clinicians (two nurses, five occupational therapists, two psychiatrists, two psychologists and two social workers). Consumers reported they were diagnosed with BPD between 1994 and 2016. Carers reported they cared for a person diagnosed between 2010 and 2023. Clinicians reported between four and 43 years of experience (mean = 19, SD = 11.2). Interview times ranged from 44 to 90 minutes (mean = 60, SD = 9.5). There were 16 interviews via Zoom and six phone calls. One interview was a consumer–carer dyad; the rest were individual. Participant quotes are coded below as CAR (carer), CON (consumer), RN (nurse), OT (occupational therapist), MD (psychiatrist), PSY (psychologist) and SW (social worker).

**TABLE 2:** Description of participants.

ID	Consumers (*n* = 4)	Sex	Year dx (pron.)	Time (minutes)
CON1	Consumer 1	Female	2016 (she/her)	53
CON2	Consumer 2	Female	1994 (she/her)	51
CON3	Consumer 3	Male	2010 (he/him)	63^a^
CON4	Consumer 4	Male	2012 (he/him)	90^b^

ID	Carers (*n* = 5)	Sex (carer)	Yr person dx (pron.)	
CAR1	Carer 1	Female	2019 (she/her)	48
CAR2	Carer 2	Female	2023 (she/her)	58^b^
CAR3	Carer 3	Female	2018 (he/him)	51
CAR4	Carer 4	Female	2018 (he/him)	58^b^
CAR5	Carer 5	Female	2010 (he/him)	63^a^

ID	Clinicians (*n* = 13)	Sex	Clinical years	
RN1	Nurse 1	Female	43	56
RN2	Nurse 2	Female	26	65
OT1	Occupational Therapist 1	Female	26	59
OT2	Occupational Therapist 2	Female	20	58
OT3	Occupational Therapist 3	Female	7	72
OT4	Occupational Therapist 4	Female	5	61
OT5	Occupational Therapist 5	Female	13	61
MD1	Psychiatrist 1	Male	30	63
MD2	Psychiatrist 2	Male	20	44^b^
PSY1	Psychologist 1	Female	25	65^b^
PSY2	Psychologist 2	Female	4	59
SW1	Social Worker 1	Female	16	70
SW2	Social Worker 2	Female	12	55^b^

Abbreviations: dx = diagnosed, pron. = pronouns, yr = year.

^a^ Dyad interview.

^b^ Phone interview.

### 3.2 Overview of themes

Five themes were generated from participants' descriptions of the daily living functioning of people diagnosed with BPD: (1) structured routines, (2) extremes of doing, (3) a lot of energy required to manage in daily life, (4) doing when disconnected from values and identity, and (5) Doing through others. Illustrative quotes are provided in Table 3.

**TABLE 3:** Illustrative quotes.

Theme 1: Structured routines		
1.1	Time in limbo: routines without purposeful, goal‐directed roles and activities	They're not doing much of anything and that leaves them feeling bored and … disconnected from the community and unhappy … They're not contributing … They're often … lonely and feel quite trapped. ^PSY1^
1.2	The structure of routines reflected in daily life	[Routines in daily life] depend on what their … external obligations are… For example, if they have to be turning up at a particular workplace at a particular time. Anything that creates structure to the day. ^MD2^ These days, I've got someone, [my baby], who completely relies on me for everything. So, I'm able to … push through things that might have previously made me just want to give up …. My day is pretty much controlled by what my 14‐month‐old is doing. ^CON1^
1.3	Stability in daily life is boring and hard	It feels really boring and you can get really resentful about having to go to bed every night at the same time or getting up every morning and trying to present yourself in the same way, whether you feel like it or not. ^OT1^ So, whilst not wishing him to become too rigid or routine focussed, I think it is important for him to recognise that routine in general is helpful, and the discipline to develop and keep a routine is very important … I'd like to see him be able to have a routine where he can get himself up in the morning and have a plan for what he's going to do that day, but this is … a big ask, given that most of his adolescent and adult life he's not had any real and meaningful ongoing routine. ^CAR4^

Theme 2: Extremes of doing		
2.1	Doing too much of one kind of domain	Some people … might be doing heaps … working full‐time, studying full‐time, and then trying to do a heap of extra things outside of that … [They're] having difficulty actually saying no to things in order to maintain their self‐care … They're not able to do things that they enjoy that gives their life a sense of fulfilment. ^OT3^
2.2	Idealising and devaluing daily activities	That idealising and devaluing of people … that happens in activities as well … They can go all in to ‘No, I'm not touching it.’ ^OT3^
2.3	Boom‐and‐bust activity patterns	You do a shitload of things and then exhaust yourself, and don't do that again for two weeks, because you need to rest … You had such an intense time… Boom‐and‐bust, like, biting off a lot because you want to … milk the opportunity when you're feeling like you can do things … and then ending up feeling exhausted. ^PSY1^ When I get into something, I can't just be happy with taking it so far, I've got to take it to the next level. And just, like, everything is over‐enhanced… I overdo things … I do things to the extreme … I was going to do this tattoo stuff… I bought all this tattoo shit, heaps of it! Not just one machine, nah fuck, I got 20 … And then… that fell in a hole, and it's all in a box unused. ^CON4^
2.4	Finding the middle ground: doing just enough	A steadiness and … a calmness about … just doing the stuff. And wouldn't it be nice if people could just be quietly proud of what they'd done, you know? Quietly proud of having a clean enough house, with … just enough laundry done … and enjoying not feeling stressed about what you have to do the next day … to stay on top of things. You know, just enough. ^OT1^

Theme 3: It takes a lot of energy to manage in daily life		
3.1	Managing strong emotions in daily life	It depends where I wake up. If I wake up on the right side of the bed, in a good mood… it's gon' be a good day … but if I get out of bed and I've dropped the shit straight away, it's gon' be a shit day, all day! ^CON2^ It depends on the quality and the mechanism of expression of the emotional dysregulation in terms of how their daily functioning gets affected. ^MD2^
3.2	Unpredictable drops in self‐care	One day, they might be absolutely okay and be able to function in their roles really quite well … And then other days, really struggle with their basic self‐care needs. ^OT2^ They can … present really well together, maybe for a month. And then, they just seem to fall apart … ‘Hang on a minute. This isn't the person that presented about a month ago …’ One week, they'll turn up presenting like they have it all together. And then… the next week, they'll come in and just go, ‘I can't do this … you're lucky I’ve even got pants on.’ ^SW1^ I don't even get out of bed some days. I just lay around in the clothes I've got on. I don't shower. I don't do nothing. ^CON2^
3.3	Constantly scanning during interactions	If you grow up from earliest childhood knowing that the world is dangerous and unpredictable, and you always need to be on the outlook for a hiding place, then this is a filter by which you assess any social interactions and creates a bias to see in relatively harmless, somewhat ambiguous behaviours the negative, and then they respond. ^MD1^
3.4	Putting on a front to survive	I put a front on people, making like, looking… like I'm doing good, but I'm not. I show the opposite. They think I'm going good but I'm not… I don't want them to see the real [me] is crashing, falling and ready to explode. ^CON2^ We are biologically wired to pick up on non‐verbal cues of emotion and expression. And so, if the non‐verbal [cues] don't match what the person is describing, the non‐verbal [cues] are going to be paid more attention to. And so, it's going to be an ineffective communication of their needs. You know, ‘I'm struggling’ or ‘I need help’ … even if they're articulating it that way, they might not appear to be struggling or need help … And so … no one sees the extent of their suffering. ^PSY2^

Theme 4: Doing things when disconnected from values and identity		
4.1	Finding something purposeful and meaningful to do	There's a lot of occupational deprivation, occupational alienation … There's really nothing that they can engage in that they enjoy … There's nothing that is meaningful to them. ^OT5^
4.2	Adopting the interests and activities of others	[Some people diagnosed with BPD] will do whatever the person they're with at the time thinks that they should do. They can be highly influenced by the people around them, because often they perceive that [the other person is] more capable and competent. And so … they'll be influenced by them. ^RN2^
4.3	Doing activities for others: relationships are important	That fragmented or incoherent sense of self can really be a determining factor, because how somebody, how anybody, decides to spend their time, is usually based on what's important to them and what their values are, and what … brings meaning into their world … [Those values] might change and fluctuate dramatically, depending on who's important to them … and who gives them a sense that they're important. So … a lot of [their daily living functioning] is relationally driven. ^OT2^ It's the relational aspect of doing things and looking for feedback from other people a lot of the time, because sometimes it's quite hard to find inherent meaning in an activity… People don't value their own sense of having done a good job… They need that relational thing of other people telling them that they've done a good job. ^OT1^

Theme 5: Doing through others		
5.1	Lost and not managing without others	She's had that experience of living by herself as well but didn't manage too well. Her mum had to go and look after her after a while. She got quite sick, both physically and mentally. She had to call the ambulance a few times and go to the hospital. So, we realised at this stage, she's not really up to living by herself. ^CAR1^
5.2	Yearning for connection	Hidden behind the rejection sensitivity is a profound yearning for connection … And so, you have this tension between a deep yearning and the inability to satisfy this. ^MD1^
5.3	Relying on others to do things	In a depressive spiral … they are physically unable to actually look after themselves and need to be cared for … They look for someone else … to take over the emotional support or physical support … If a person with BPD has a close contact, usually family, but sometimes their partner … they will transfer these [daily] activities and responsibilities through to their loved ones. ^MD2^
5.4	Shifting roles in relationships	When she's upset … her partner just does all these things … He is more likely to get more active, make offers to help … And in the short term, it's absolutely such a relief, and a very supportive thing. And in the longer term, I don't think it serves her. ^PSY1^

### 3.3 Theme 1: Structured routines

Theme 1 has three subthemes, describing the importance of structured routines for people diagnosed with BPD.

#### 3.3.1 Subtheme 1.1—Time in limbo: Routines without purposeful, goal‐directed roles and activities

Consumers described routines without purposeful, goal‐directed roles and activities as being ‘*in limbo*’ ^CON1^; ‘*sitting here doing nothing*’ ^CON2^; ‘*sitting back, smoking cones*’ ^CON3^; and ‘*laying on me bed, doing not much at all*’ ^CON4^. Carers mirrored these descriptions: ‘*he gets bored… he feels lost and aimless*’ ^CAR4^; ‘*she just [does] nothing*’ ^CAR2^; and ‘*he sits around in his bedroom all day … not really doing very much*’ ^CAR3^.

#### 3.3.2 Subtheme 1.2—The structure of routines reflected in daily life

Routines without structure led to ‘*waking up and responding to whatever comes through their phone*’ ^OT3^. There were inconsistent or late sleep patterns, with one carer describing how her person ‘*spends a lot of time sleeping during the day, and then she is more active at night*’ ^CAR1^. A consumer reported: ‘*Sleep is my big goal in life… I crave the sleep… I've always had trouble sleeping*’ ^CON1^. Nighttime routines were likewise unstructured. One consumer described ‘*the quietness of everything at night. Everyone else is sleeping, and I can be awake*’ ^CON4^. However, nighttime was also deemed ‘*the riskiest time*’ ^SW1^. The night could also involve being ‘*up gaming … watching movies … getting onto social media and scrolling for as long as they can*’ ^RN1^. Nutritional intake was also reflected in unstructured routines, with ‘*a lot of junk food, fast food, easy foods*’ ^RN1^.

Inversely, when people diagnosed with BPD found purposeful and goal‐directed roles and activities to structure their routines, there were positive flow‐on effects across multiple domains of daily living, including ‘*regular self‐care … brushing their teeth, making regular meals, getting dressed, planning their day*’ ^OT3^. Examples of purposeful roles included work, study, volunteering, parenting, household management, attending psychotherapy, hobbies and pet care. ‘*Working can be a great containment for a person because they have to get up … show up … [and] be presentable*’ ^OT5^.

#### 3.3.3 Subtheme 1.3—Stability in daily life is boring and hard

Although structured routines were described as important, people diagnosed with BPD often struggled to establish such routines because ‘*it feels really boring*’ ^OT1^. Moreover, they could have ‘*difficulty tolerating [activities] that are not interesting*’ ^OT3^. A structured routine could also lead to feelings of resentment ^OT1^.

### 3.4 Theme 2: Extremes of doing

Theme two has four subthemes, describing how daily living functioning was characterised by extremes of doing.

#### 3.4.1 Subtheme 2.1—Doing too much of one kind of domain

People diagnosed with BPD were often ‘*trying to do it all … and feeling overwhelmed and exhausted and burnt out*’ ^PSY1^ in the process. Doing too much could mean an over‐emphasis on one domain of functioning, such as studying, working or parenting full time but then neglecting self‐care, leisure or relationships. Clinicians described a natural proclivity for creativity. However, creativity and leisure pursuits could become their only activity ‘*for large chunks of the day*’ ^OT3^, to the exclusion of other activities such as medication ^CAR1^ or household ^OT4^ management.

#### 3.4.2 Subtheme 2.2—Idealising and devaluing daily activities

Daily living functioning was described as ‘*either … all good or all crap. There doesn't seem to be a happy medium*’ ^RN1^. This dichotomous way of doing was underpinned by likewise ‘*out of balance*’ ^PSY1^ self‐expectations: ‘“*I've got to be doing this, this and this, and it's got to be absolutely perfect … or… I'm just incapable of doing anything*”’ ^PSY1^. They could swing between ‘“*I really love doing this art’ and then, ‘I'm terrible. That's really boring*”’ ^OT3^.

#### 3.4.3 Subtheme 2.3—Boom‐and‐bust activity patterns

Daily living functioning could follow ‘*boom‐and‐bust*’ ^PSY2^ activity patterns, that is, a pattern of doing too much followed by withdrawal from activity (see Section 3.3.1). A ‘boom’ in activity arose from brief windows of ‘*feeling like you can do things*’ ^PSY1^. People diagnosed with BPD may ‘*do things to the extreme*’ ^CON4^ and ‘*be really enthusiastic … jump all the way in*’ ^OT3^, starting new hobbies, courses or jobs. Then, they will ‘bust’ or ‘*withdraw completely, rather than negotiate how to make it sustainable*’ ^OT3^.

#### 3.4.4 Subtheme 2.4—Finding the middle ground: doing just enough

People diagnosed with BPD had difficulties finding the ‘middle ground’ in daily living functioning, as they were either ‘*filling their lives with lots of different things to keep them[selves] out of their own mind*’ ^PSY2^ or experiencing complete withdrawal. Thus, they might benefit from support ‘*to be able to live a slower life that isn't so chaotic*’ ^SW2^ and to be doing ‘*just enough*’ ^OT1^.

### 3.5 Theme 3: It takes a lot of energy to manage in daily life

Theme three has four subthemes, describing how managing strong emotions and navigating interpersonal interactions could deplete energy needed for daily living functioning.

#### 3.5.1 Subtheme 3.1—Managing strong emotions in daily life

Strong emotions shaped the daily living functioning of people diagnosed with BPD ‘*and that's where the daily functioning can be impaired*’ ^MD2^. Their ability to perform daily activities was ‘*not so much in terms of cognitive things*’ ^MD2^. Rather, it was strong emotions that prevented execution, such that ‘*they might have the skills to cook and clean, but … it's too overwhelming*’ ^OT4^. Strong emotions could ‘*throw them off quickly*’ ^RN2^, making it ‘*very hard for them to shift their attention to …* “*I need to be at the bus stop at this time*” *or* “*I need to pay the bill today*”’ ^RN2^. ‘*The intensity of that emotional experience*’ ^RN2^ ‘*can quite quickly cause that withdrawal*’ ^OT3^, creating ‘*urges to avoid or delay tasks*’ ^RN2^.

Daily living functioning shaped by strong emotions was also described as ‘*doing only the easy things that gratify quickly, while putting off daily tasks and responsibilities that are more relevant to … long‐term goals*’ ^PSY1^, such as ‘*making an appointment with the doctor*’ ^PSY1^. People could stop taking their medications ‘*driven by not feeling like taking them*’ ^SW1^ or not turn up to appointments (or interviews for this study): ‘*I get close to that date. Nup, it's cancelled*’ ^CON2^.

People diagnosed with BPD could use illicit substances to provide ‘*subjective relief of their emotional states*’ ^MD2^, though this relief was temporary. Marijuana use was described as ‘*the only way I can sleep*’ ^CON2^. However, marijuana also ‘*keeps the [BPD] symptoms going*’ ^MD2^. Methamphetamine use made ‘*most areas of functioning more difficult*’ ^OT3^. No matter the coping style, managing strong emotions in daily life was exhausting.

#### 3.5.2 Subtheme 3.2—Unpredictable drops in self‐care

One carer described her person's rapid changes in emotional states: ‘*it would just take one thing just to… set it off … One minute, he's happy. Next minute, he's just in his grumpy mood*’ ^CAR5^. During an ‘*emotionally activated*’ ^OT2^, ‘*bad day*’ ^CON2^, ‘*self‐care was one of the first things to drop off*’ ^OT3^. Emotional unpredictability meant that people diagnosed with BPD could not ‘*take it for granted*’ ^RN2^ when their emotional states were ‘*going smoothly*’ ^RN2^, and this trepidation drained energy in daily life.

#### 3.5.3 Subtheme 3.3—Constantly scanning during interactions

Navigating interpersonal interactions placed another substantial burden on energy. A consumer said: ‘*What affects my life from day to day, sort of thing? The interactions, you know … it doesn't take much for someone to really trigger me*’ ^CON4^. A nurse reported people diagnosed with BPD were ‘*constantly … in that hypervigilance mode*’ ^RN1^, which meant they were ‘*never truly present … always trying to analyse what's just been said and respond*’ ^RN1^. A psychiatrist explained: ‘*being on guard in relationships is very exhausting*’ ^MD1^.

#### 3.5.4 Subtheme 3.4—Putting on a front to survive

People diagnosed with BPD ‘*try and keep it all together*’ ^OT2^ by performing ‘*masking*’ ^PSY2^, ‘*apparent competence*’ ^SW1^ or putting ‘*on a front*’ ^CON2^, that is, presenting an orderly exterior while managing internal chaos. This meant they were ‘*good at surviving*’ ^PSY2^ because they could adapt ‘*who they are to fit in with the people around them*’ ^OT2^. However, this also meant that ‘*no one sees the extent of their suffering*’ ^PSY2^. The energy required to put up and maintain such fronts was unsustainable.

### 3.6 Theme 4: Doing things when disconnected from values and identity

Theme four has three subthemes, describing how disconnection from values and identity impacts the daily living functioning of people diagnosed with BPD.

#### 3.6.1 Subtheme 4.1—Finding something purposeful and meaningful to do

An occupational therapist said: ‘*how anybody decides to spend their time is usually based on what's important to them … what their values are*’ ^OT2^. However, a ‘*fragmented or incoherent sense of self*’ ^OT2^ could lead to challenges with finding purpose and meaning in daily activities. One consumer stated: ‘*nothing's really important to me*’ ^CON4^. Their ‘*struggle to identify what they're interested in*’ ^PSY2^ meant ‘*they just don't have anything that actually sparks any joy in their life*’ ^OT2^ or ‘*they might … jump around between things*’ ^PSY2^.

Without core values underpinning decision‐making, people diagnosed with BPD may seek meaning through self‐defeating activities, such as ‘*dangerous driving*’ ^MD2^, ‘*promiscuity*’ ^RN1^ or ‘*impulsive spending, lots of online shopping*’ ^SW2^. They may also struggle to ‘*recognise achievements*’ ^RN1^ or ‘*they can brush it off*’ ^SW1^, which could ‘*stem from the fact that they have often never been praised for any achievement in their lives*’ ^RN1^. Identifying core values ‘*takes a long time … months and months of work*’ ^OT2^. However, ‘*the biggest adjustments are made*’ ^OT2^ to daily living functioning ‘*when there's a values‐orientation driving it*’ ^OT2^. A nurse also described the importance of ‘*self‐compassion … having that sense of being worthy enough to make decisions about their lives, and to dictate their own day*’ ^RN2^.

#### 3.6.2 Subtheme 4.2—Adopting the interests and activities of others

Without identified and clarified core values and a stable sense of self to guide decision‐making in daily life, people diagnosed with BPD ‘*can be highly influenced by the people around them*’ ^RN2^. They could ‘*do whatever the person they're with at the time thinks that they should do*’ ^RN2^. Attempts to engage in their own interests could ‘*drop off quickly … if people in their social circle … don't value that [interest] the same*’ ^OT3^. Social media also played a role: ‘*they might grab hold of an influencer for a while … [and] get very attached to different ideas*’ ^SW1^. Adopting the activities of others could also lead to ‘*negative situations*’ ^CAR4^, including being around others who use illicit substances: ‘*The more I go there, the more drugs I get on!*’ ^CON2^.

#### 3.6.3 Subtheme 4.3—Doing activities for others: Relationships are important

The daily living functioning of people diagnosed with BPD was characterised as ‘*relationally driven*’ ^OT2^ through ‘*constantly looking for that feedback from others to validate who they are as a person*’ ^RN1^. One consumer described meal planning in terms of liking ‘*people to be proud of me*’ ^CON1^. Another expressed doing for others as ‘*the reason why I'm here … if I didn't have my grandkids, I wouldn't be alive*’ ^CON2^. Yet, doing for others could become ‘*quite codependent … she spends most of her day driving her partner to and from work, and waiting for him, and doing the housework*’ ^RN2^. A psychologist said: ‘*it would be way easier for someone with BPD to do for others than it would be to do the exact same thing for themselves*’ ^PSY1^.

### 3.7 Theme 5: Doing through others

Theme five has four subthemes, describing how the daily living functioning of people diagnosed with BPD could involve doing through others, that is, the relational aspects of engagement in activities.

#### 3.7.1 Subtheme 5.1—Lost and not managing without others

Clinicians described how people diagnosed with BPD ‘*hate to be alone*’ ^PSY1^; ‘*if there's no one around them, they can be very lost*’ ^SW1^. Carers mirrored these descriptions: ‘*he needs to be with others and hates to be alone*’ ^CAR4^. Attempts to live alone were described by carers as ‘*an absolute disaster*’ ^CAR3^ because they ‘*need to be with other people*’ ^CAR1^.

#### 3.7.2 Subtheme 5.2—Yearning for connection

People diagnosed with BPD were described as having ‘*a profound yearning for connection*’ ^MD1^, which some participants attributed to a ‘*deprived childhood*’ ^SW2^, where they may have ‘*been traumatised, abandoned [and] neglected*’ ^CAR4^. A social worker described how one person ‘*was trying to connect with people but just couldn't quite work out how to do it in an effective and safe way*’ ^SW2^. In relationships, people could experience ‘*loving that person but hating them at the same time*’ ^RN1^; polarised positions that could disrupt daily living functioning. A consumer said: ‘*I had to stop working because I just couldn't connect with people properly*’ ^CON1^.

#### 3.7.3 Subtheme 5.3—Relying on others to do things

A psychiatrist reported that the daily living functioning of people diagnosed with BPD was subject to a ‘*depressive spiral*’ ^MD2^ wherein they ‘*transfer responsibilities*’ ^MD2^ and become reliant on others. As one consumer described:

I tend to, kind of, downward spiral … things [that] I normally manage fine … simple things, like getting the food shopping delivered instead of going and getting it … making my husband come home from work early so he can pick the kids up from school … because I just can't … I'm just too overwhelmed. ^CON1^

Carers' descriptions of relying on others for daily living functioning included: being woken up ^CAR4^, managing medications ^CAR1^, preparing meals ^CAR3^, providing transport ^CAR1^, doing the shopping ^CAR3^ and managing finances ^CAR2^.

#### 3.7.4 Subtheme 5.4—Shifting roles in relationships

Relying on others for daily living functioning could lead to shifts in relationship roles. Romantic partnerships could fall into a pattern where ‘*the [partner] is taking care of them*’ ^PSY1^. One carer reported being with her person ‘*24/7, apart from when he's with [his support worker]*’ ^CAR5^. Problems with stepping back from ‘active’ support by partners was attributed to ‘*fear … ‘What if that causes an escalation … frequent admissions to hospital [and] self‐harm?’*’ ^OT4^. However, this also led to shifts in relationship roles, meaning ‘*they no longer call each other even their partner. They'll say, ‘We're living under the same roof, but I'm just a carer now.’ So, the intimacy of the relationship goes*’ ^OT4^. Then, problems with intimacy remained unaddressed because ‘*sex is often a fraught topic … even talking about it brings up a lot of shame and fear and disgust*’ ^PSY1^.

## 4 DISCUSSION

This qualitative study described the daily living functioning of people diagnosed with BPD from consumer, carer and clinician perspectives. Five themes were generated that highlighted the importance of: (1) structuring routines through purposeful roles and activities; (2) maintaining balance across multiple domains of daily living; (3) managing energy depleted by emotional intensity and interpersonal difficulties; (4) connecting with core values to derive meaning from roles and activities; and (5) understanding the extent of carer involvement in performing daily activities. These findings have implications for understanding daily living functioning beyond a single score on the Global Assessment of Functioning Scale and ‘work and love’ milestones (Culina et al., 2024; Desrosiers et al., 2020; Dhar et al., 2023; Endicott et al., 1976; Grenyer et al., 2022; Storebø et al., 2020). Findings could also be used to develop BPD‐specific outcome measures and rehabilitation programs to enable functional recovery (Desrosiers et al., 2017, 2026; Tepper et al., 2025). Occupational therapists could be at the forefront of supporting functional recovery outcomes for this population (Weideman, 2025).

Participants in this study described sleep challenges in terms of being awake at night and then sleeping (or doing passive activities) during the day. Such descriptions were consistent with a BPD‐specific circadian chronotype (McGowan & Saunders, 2021; Winsper et al., 2017). Disorganised sleep patterns could contribute to difficulties establishing structured routines in daily life. Currently, the only major psychotherapy that specifically targets sleep is DBT, which sees sleep as one of many ‘emotional regulation’ skills (Linehan, 2015, pp. 397–401). This study affirms the need for occupational therapists to address sleep as a core aspect of daily living functioning for people diagnosed with BPD (Ho & Siu, 2018; Jenkins et al., 2022; King et al., 2024; van Trigt et al., 2025; Wood et al., 2015).

Descriptions of activity pacing and energy management problems in this study were consistent with terminology from chronic pain literature (Andrews et al., 2021, 2012). Emotional pain might have a similar impact on daily living functioning as physical pain, which is interesting given the well‐established complex interplay between BPD, chronic pain and fatigue (Álvarez‐Tomás et al., 2024; Nance et al., 2024). Thus, it seems possible that chronic disease models could be adapted to support the functional recovery needs of people diagnosed with BPD (Grover & Joshi, 2014). Activity pacing strategies, such as setting agendas, taking breaks and maintaining life balance, are already described in the BPD literature to maintain vocational functioning (Larivière et al., 2025). Similar strategies could also be applied to other domains of daily living, such as self‐care, routines and sleep.

This research found descriptions of people diagnosed with BPD having problems with deriving intrinsic meaning from roles and daily activities, which may be attributed to a ‘fragmented sense of self’ and disconnection from values (Cameron et al., 2014; Faggioli et al., 2024; Kaufman & Meddaoui, 2021; Mohi et al., 2018). Problems forming a coherent self‐concept could be attributed to adverse childhood experiences, that is, unmet emotional needs within invalidating environments and/or disrupted early attachment relationships (Bateman & Fonagy, 2004; de Aquino Ferreira et al., 2018; Estric et al., 2022; Linehan, 1993; Yeomans et al., 2015; Young et al., 2003). A psychotherapeutic approach to identity formation might entail developing psychological ‘skills’ or ‘insights’. An occupation‐based approach could involve supporting persistence in a self‐selected hobby or creative project (Borovica et al., 2024; Goodman, 1983; Ludowyke et al., 2024). The considerable work to support values clarification holds promise for improving functional outcomes.

People diagnosed with BPD were described by participants as having unpredictable and fluctuating emotional states (Chapman, 2019; D'Aurizio et al., 2023). During ‘emotional activation’, the responsibility for performing daily activities could be ‘transferred’ onto carers. This dynamic may be underpinned by carers' fear of self‐harm or emotional escalation, driven by unmet ‘implicit dependency needs’ (Bornstein et al., 2010). However, over time, this dynamic might lead to longer‐term shifts in relationship roles (i.e., from partner to carer). The person then has less opportunity for improving their functional capacity in daily activities or developing confidence and identity through occupational engagement (Hansson et al., 2022). Therefore, where appropriate and consented, rehabilitation for daily living functioning could adopt a family‐centred approach, which could support the restoration or maintenance of relationship roles (Guillen et al., 2021).

### 4.1 Recommendations for future research

Several key themes warrant further research. For instance, evaluating the effectiveness and feasibility of BPD‐specific sleep interventions (King et al., 2024). Research could also investigate whether activity pacing is a valid construct for BPD and if principles of chronic disease models apply in a rehabilitation framework for this population (Andrews et al., 2012; Grover & Joshi, 2014). Given the breadth of domains affected, comprehensive BPD‐specific outcome measures are needed to evaluate changes in functioning across multiple domains of daily living (Desrosiers et al., 2017, 2026; Dhar et al., 2023; Tepper et al., 2025). Outcome measures could include the structure of routines, activity pacing, life balance, energy management, values identification and the extent of carer involvement in daily activities, which could potentially underpin the evidence base of a rehabilitation program (Desrosiers et al., 2017, 2026; Tepper et al., 2025).

### 4.2 Relevance for clinical practice

The findings of this study provide a perspective on daily living functioning for people diagnosed with BPD beyond a psychotherapeutic framework or a focus on vocational and relational domains. Occupational therapists need to prioritise *consistent sleep patterns* when working with people diagnosed with BPD, which might include DBT sleep protocols and weighted blankets (Ho & Siu, 2018; King et al., 2024; Linehan, 2015; Steingrímsson et al., 2022; van Trigt et al., 2025).

*Activity pacing* interventions from chronic pain approaches could be adapted to address apparent ’boom‐and‐bust’ activity patterns (Andrews et al., 2012). Strategies used to maintain vocational functioning include structuring tasks, setting alarms and taking breaks (Larivière et al., 2025). Similar strategies could also be applied to other domains of daily living.

Occupational therapists could explore *values and identity* for people diagnosed with BPD (Cameron et al., 2014; Hansson et al., 2022; Mohi et al., 2018). There is scope to support *persistence* with a self‐selected, activity‐based project as a means of supporting this process (Goodman, 1983). Where consented and appropriate, the extent of carer involvement in daily activities must be explored (Guillen et al., 2021).

### 4.3 Limitations

There were limitations to this study, including the sample being clinician‐heavy (13 clinicians compared to four consumers and five carers). This discrepancy arose because of challenges with recruitment; greater inclusion of these voices would have strengthened findings. During analysis, the research team used reflexivity and took an inductive approach to managing the data. The data were synthesised such that themes included the voices of consumers and carers alongside clinicians where possible. Effort was made to ensure that there was a spread of quotes used in the data reporting from across the three cohorts. The consumer and carer cohorts were recruited based on self‐report of a BPD diagnosis. Although member checking was undertaken, there was only one participant who accepted the invitation and took the opportunity to provide feedback. The sample was limited to Australian participants and may not reflect other cultural contexts. Recruitment through social media and professional networks potentially attracted informed or proactive participants, introducing self‐selection bias. The interviews relied on retrospective accounts and perceptions, which may be subject to recall bias or be influenced by current mood states.

## 5 CONCLUSION

This qualitative study explored the perspectives of 22 participants (consumers, carers and clinicians) about the daily living functioning of people diagnosed with BPD. Five themes highlighted the urgent need to address daily living functioning for this population. Longitudinal research consistently demonstrates persistent functional impairment, which a psychotherapeutic approach alone does not necessarily ameliorate. Rehabilitation targets in occupational therapy could include establishing structure in routines and sleep patterns. People diagnosed with BPD could benefit from activity pacing strategies (a chronic pain approach) to manage the impacts of emotional pain on daily living functioning. Support to clarify a person's core values shows promise for finding purpose and meaning in daily activities. The extent and nature of carer involvement in daily activities must be explored so that people diagnosed with BPD can reap the identity formation rewards of occupational performance.

## AUTHOR CONTRIBUTIONS

Dillon Tepper: investigation, analysis and interpretation, drafting of manuscript, project administration. Ben Sellar: conception and methodology, analysis and interpretation, drafting and critical review of manuscript, supervision. Sheryl Shipley: drafting and critical review of manuscript, supervision. Rachel Smith: drafting and critical review of manuscript, supervision. Carolyn M Murray: conception and methodology, analysis and interpretation, drafting and critical review of manuscript, supervision. All authors listed meet the authorship criteria according to the latest guidelines of the International Committee of Medical Journal Editors. All authors have read and agree with the final manuscript.

## CONFLICT OF INTEREST STATEMENT

The authors have no conflict of interest to declare. Co‐author Dr Carolyn Murray is an associate editor for the Australian Occupational Therapy Journal and was not involved in the handling of this manuscript.

## ETHICS APPROVAL STATEMENT

The University of South Australia Human Research Ethics Committee provided approval for this study (206296).

## CONSENT

Consent for publication was obtained as part of written informed consent for this study.

## DECLARATION OF USE OF ARTIFICIAL INTELLIGENCE

Claude (Sonnet 4.5, Anthropic) on private settings was used for checking grammar and reference accuracy to meet the standards expected by the journal. AI was not used to conceptualise the research, generate text or analyse data.

## Data Availability

Research data are not shared.

## References

[aot70127-bib-0001] Álvarez‐Tomás, I. , Ruiz, J. , Guilera, G. , & Bados, A. (2019). Long‐term clinical and functional course of borderline personality disorder: A meta‐analysis of prospective studies. European Psychiatry, 56(1), 75–83. 10.1016/j.eurpsy.2018.10.010 30599336

[aot70127-bib-0002] Álvarez‐Tomás, I. , Soler, J. , Schmidt, C. , & Pascual, J. C. (2024). Physical health, primary care utilization and long‐term quality of life in borderline personality disorder: A 10‐year follow‐up study in a Spanish sample. Journal of Psychosomatic Research, 179, 111623. 10.1016/j.jpsychores.2024.111623 38422718

[aot70127-bib-0003] American Psychiatric Association . (2013). Diagnostic and Statistical Manual of Mental Disorders (5th ed.). American Psychiatric Publishing. 10.1176/appi.books.9780890425596

[aot70127-bib-0004] Andrews, N. E. , Chien, C. W. , Ireland, D. , & Varnfield, M. (2021). Overactivity assessment in chronic pain: The development and psychometric evaluation of a multifaceted self‐report assessment. European Journal of Pain, 25(1), 225–242. 10.1002/ejp.1664 PMC7821341 32965735

[aot70127-bib-0005] Andrews, N. E. , Strong, J. , & Meredith, P. J. (2012). Activity pacing, avoidance, endurance, and associations with patient functioning in chronic pain: A systematic review and meta‐analysis. Archives of Physical Medicine and Rehabilitation, 93(11), 2109–2121. 10.1016/j.apmr.2012.05.029 22728699

[aot70127-bib-0006] Aslam, H. M. , Brodeur, J. , & Links, P. S. (2025). Clinical practice guidelines for the treatment of borderline personality disorder: A systematic review of best practice in anticipation of MAiD MD‐SUMC. Borderline Personality Disorder and Emotion Dysregulation, 12(1), 1–13. 10.1186/s40479-025-00284-5 PMC11983944 40211336

[aot70127-bib-0007] Bateman, A. , & Fonagy, P. (2004). Mentalization‐based treatment of BPD. Journal of Personality Disorders, 18(1), 36–51. 10.1521/pedi.18.1.36.32772 15061343

[aot70127-bib-0008] Birken, M. , & Harper, S. (2017). Experiences of people with a personality disorder or mood disorder regarding carrying out daily activities following discharge from hospital. British Journal of Occupational Therapy, 80(7), 409–416. 10.1177/0308022617697995

[aot70127-bib-0009] Bornstein, R. F. , Becker‐Matero, N. , Winarick, D. J. , & Reichman, A. L. (2010). Interpersonal dependency in borderline personality disorder: Clinical context and empirical evidence. Journal of Personality Disorders, 24(1), 109–127. 10.1521/pedi.2010.24.1.109 20205501

[aot70127-bib-0010] Borovica, T. , Kokanović, R. , Seal, E.‐L. , Flore, J. , Boydell, K. , Blackman, L. , & Hayes, L. (2024). What does leisure have to do with mental health–arts, creative and leisure practices and living with mental distress. Leisure Studies, 45, 1–15. 10.1080/02614367.2024.2376016

[aot70127-bib-0011] Braun, V. , & Clarke, V. (2022). Thematic analysis: A practical guide. SAGE Publications Ltd.

[aot70127-bib-0012] Burr, V. , & Dick, P. (2017). Social constructionism. In B. Gough (Ed.), The Palgrave handbook of critical social psychology (pp. 59–80). Palgrave Macmillan UK. 10.1057/978-1-137-51018-1_4

[aot70127-bib-0013] Cameron, A. Y. , Palm Reed, K. , & Gaudiano, B. A. (2014). Addressing treatment motivation in borderline personality disorder: Rationale for incorporating values‐based exercises into dialectical behavior therapy. Journal of Contemporary Psychotherapy, 44, 109–116. 10.1007/s10879-013-9253-9

[aot70127-bib-0014] Chapman, A. L. (2019). Borderline personality disorder and emotion dysregulation. Development and Psychopathology, 31(3), 1143–1156. 10.1017/S0954579419000658 31169118

[aot70127-bib-0015] Choi‐Kain, L. W. , Finch, E. F. , Masland, S. R. , Jenkins, J. A. , & Unruh, B. T. (2017). What works in the treatment of borderline personality disorder. Current Behavioral Neuroscience Reports, 4(1), 21–30. 10.1007/s40473-017-0103-z PMC5340835 28331780

[aot70127-bib-0016] Choi‐Kain, L. W. , Reich, D. B. , Masland, S. R. , Iliakis, E. A. , & Ilagan, G. S. (2020). Longitudinal course of borderline personality disorder: What every clinician needs to know. Current Treatment Options in Psychiatry, 7(3), 429–445. 10.1007/s40501-020-00223-x

[aot70127-bib-0017] Culina, I. , Ranjbar, S. , Maillard, P. , Martin‐Soelch, C. , Berney, S. , Kolly, S. , André, J. , Conus, P. , & Kramer, U. (2024). Symptom domains and psychosocial functioning in borderline personality disorder. Borderline Personality Disorder and Emotion Dysregulation, 11(1), 10. 10.1186/s40479-024-00255-2 PMC11151627 38835094

[aot70127-bib-0018] D'Aurizio, G. , Di Stefano, R. , Socci, V. , Rossi, A. , Barlattani, T. , Pacitti, F. , & Rossi, R. (2023). The role of emotional instability in borderline personality disorder: A systematic review. Annals of General Psychiatry, 22(1), 9. 10.1186/s12991-023-00439-0 PMC10011773 36918920

[aot70127-bib-0019] de Aquino Ferreira, L. F. , Pereira, F. H. Q. , Benevides, A. M. L. N. , & Melo, M. C. A. (2018). Borderline personality disorder and sexual abuse: A systematic review. Psychiatry Research, 262, 70–77. 10.1016/j.psychres.2018.01.043 29407572

[aot70127-bib-0020] Desrosiers, J. , Larivière, N. , Desrosiers, J. , Boyer, R. , David, P. , Bérubé, F. A. , & Pérusse, F. (2017). Conception of a daily functioning assessment tool for people with borderline personality disorder, the Functional Assessment for Borderline personality disorder (FAB). Revue Francophone de Recherche en Ergothérapie, 3(2), 17–38. 10.13096/rfre.v3n2.77

[aot70127-bib-0021] Desrosiers, J. J. , Bertrand Leiser, A. M. , Giguère, C.‐E. , & Cailhol, L. (2026). Psychometric properties of the FAB2 for borderline personality disorder: A Swiss validation. Occupational Therapy in Mental Health, 1–18. 10.1080/0164212X.2026.2685306

[aot70127-bib-0022] Desrosiers, J. J. , Larivière, N. , Desrosiers, J. , & Boyer, R. (2020). Concept analysis of human functioning and potential applications to mental health evaluation. Occupational Therapy in Mental Health, 36(1), 1–28. 10.1080/0164212X.2019.1684860

[aot70127-bib-0023] Dhar, A. , Hay, P. , & Meade, T. (2023). Social and occupational functioning in individuals with BPD: A systematic review. Journal of Personality Disorders, 37(6), 691–723. 10.1521/pedi.2023.37.6.691 38038659

[aot70127-bib-0024] Dixon, L. J. , & Linardon, J. (2020). A systematic review and meta‐analysis of dropout rates from dialectical behaviour therapy in randomized controlled trials. Cognitive Behaviour Therapy, 49(3), 181–196. 10.1080/16506073.2019.1620324 31204902

[aot70127-bib-0025] Endicott, J. , Spitzer, R. L. , Fleiss, J. L. , & Cohen, J. (1976). The Global Assessment Scale: A procedure for measuring overall severity of psychiatric disturbance. Archives of General Psychiatry, 33(6), 766–771. 10.1001/archpsyc.1976.01770060086012 938196

[aot70127-bib-0026] Estric, C. , Calati, R. , & Lopez‐Castroman, J. (2022). Adverse childhood experiences and neurocognition in borderline personality disorder: A call‐to‐action perspective review. Harvard Review of Psychiatry, 30(4), 248–260. 10.1097/HRP.0000000000000344 35849742

[aot70127-bib-0027] Faggioli, I. , Esposito, C. M. , & Stanghellini, G. (2024). Identity and temporal fragmentation in borderline personality disorder: A systematic review. Brain Sciences, 14(12), 1221. 10.3390/brainsci14121221 PMC11727200 39766420

[aot70127-bib-0028] Gartlehner, G. , Crotty, K. , Kennedy, S. , Edlund, M. J. , Ali, R. , Siddiqui, M. , Fortman, R. , Wines, R. , Persad, E. , & Viswanathan, M. (2021). Pharmacological treatments for borderline personality disorder: A systematic review and meta‐analysis. CNS Drugs, 35, 1053–1067. 10.1007/s40263-021-00855-4 PMC8478737 34495494

[aot70127-bib-0029] Goodman, G. B. (1983). Occupational therapy treatment: Interventions with borderline patients. Occupational Therapy in Mental Health, 3(3), 19–31. 10.1300/J004v03n03_02

[aot70127-bib-0030] Grenyer, B. F. , Townsend, M. L. , Lewis, K. , & Day, N. (2022). To love and work: A longitudinal study of everyday life factors in recovery from borderline personality disorder. Personality and Mental Health, 16(2), 138–154. 10.1002/pmh.1547 PMC9287094 35538561

[aot70127-bib-0031] Grover, A. , & Joshi, A. (2014). An overview of chronic disease models: A systematic literature review. Global Journal of Health Science, 7(2), 210–227. 10.5539/gjhs.v7n2p210 PMC4796376 25716407

[aot70127-bib-0032] Guillen, V. , Diaz‐Garcia, A. , Mira, A. , Garcia‐Palacios, A. , Escrivá‐Martínez, T. , Banos, R. , & Botella, C. (2021). Interventions for family members and carers of patients with borderline personality disorder: A systematic review. Family Process, 60(1), 134–144. 10.1111/famp.12537 32304101

[aot70127-bib-0033] Gunderson, J. G. , Stout, R. L. , McGlashan, T. H. , Shea, M. T. , Morey, L. C. , Grilo, C. M. , Zanarini, M. C. , Yen, S. , Markowitz, J. C. , & Sanislow, C. (2011). Ten‐year course of borderline personality disorder psychopathology and function from the Collaborative Longitudinal Personality Disorders study. Archives of General Psychiatry, 68(8), 827–837. 10.1001/archgenpsychiatry.2011.37 PMC3158489 21464343

[aot70127-bib-0034] Hansson, S. O. , Björklund Carlstedt, A. , & Morville, A.‐L. (2022). Occupational identity in occupational therapy: A concept analysis. Scandinavian Journal of Occupational Therapy, 29(3), 198–209. 10.1080/11038128.2021.1948608 34260864

[aot70127-bib-0035] Ho, E. C. , & Siu, A. M. (2018). Occupational therapy practice in sleep management: A review of conceptual models and research evidence. Occupational Therapy International, 2018(1), 8637498. 10.1155/2018/8637498 PMC6087566 30150906

[aot70127-bib-0036] Iliakis, E. A. , Ilagan, G. S. , & Choi‐Kain, L. W. (2021). Dropout rates from psychotherapy trials for borderline personality disorder: A meta‐analysis. Personality Disorders, Theory, Research, and Treatment, 12(3), 193–206. 10.1037/per0000453 33591777

[aot70127-bib-0037] Jenkins, C. A. , Thompson, K. N. , Nicholas, C. L. , & Chanen, A. M. (2022). Sleep in young people with features of borderline personality disorder: A scoping review. Journal of Personality Disorders, 36(1), 19–39. 10.1521/pedi_2021_35_525 34124946

[aot70127-bib-0038] Kaufman, E. A. , & Meddaoui, B. (2021). Identity pathology and borderline personality disorder: An empirical overview. Current Opinion in Psychology, 37, 82–88. 10.1016/j.copsyc.2020.08.015 32932110

[aot70127-bib-0039] King, J. D. , Cheng, S. , Fok, M. L. Y. , Pappa, S. , & Munjiza, J. (2024). Interventions to improve the sleep quality of adults with personality disorder: A systematic review. Personality and Mental Health, 18(1), 19–31. 10.1002/pmh.1591 37674476

[aot70127-bib-0040] Klein, P. , Fairweather, A. K. , & Lawn, S. (2022). Structural stigma and its impact on healthcare for borderline personality disorder: A scoping review. International Journal of Mental Health Systems, 16(1), 48. 10.1186/s13033-022-00558-3 PMC9520817 36175958

[aot70127-bib-0041] Lak, M. , Shakiba, S. , Dolatshahi, B. , Saatchi, M. , Shahrbaf, M. , & Jafarpour, A. (2025). The prevalence of suicide ideation, suicide attempt and suicide in borderline personality disorder patients: A systematic review and meta‐analysis. General Hospital Psychiatry, 95, 52–61. 10.1016/j.genhosppsych.2025.04.005 40279864

[aot70127-bib-0042] Larivière, N. , Corbière, M. , Robitaille‐Beaumier, E.‐L. , David, P. , & Cailhol, L. (2025). What strategies do people with borderline personality disorder use to maintain their well‐being and performance at work? Borderline Personality Disorder and Emotion Dysregulation, 12(1), 18. 10.1186/s40479-025-00293-4 PMC12080128 40369685

[aot70127-bib-0043] Larivière, N. , Denis, C. , Payeur, A. , Ferron, A. , Levesque, S. , & Rivard, G. (2016). Comparison of objective and subjective life balance between women with and without a personality disorder. Psychiatric Quarterly, 87(4), 663–673. 10.1007/s11126-016-9417-3 26875106

[aot70127-bib-0044] Leichsenring, F. , Fonagy, P. , Heim, N. , Kernberg, O. F. , Leweke, F. , Luyten, P. , Salzer, S. , Spitzer, C. , & Steinert, C. (2024). Borderline personality disorder: A comprehensive review of diagnosis and clinical presentation, etiology, treatment, and current controversies. World Psychiatry, 23(1), 4–25. 10.1002/wps.21156 PMC10786009 38214629

[aot70127-bib-0045] Linehan, M. (1993). Cognitive‐behavioral treatment of borderline personality disorder. Guilford Press.

[aot70127-bib-0046] Linehan, M. M. (2015). DBT skills training manual. Guilford Press.

[aot70127-bib-0047] Louise, S. , Dondzilo, L. , Padmanabhan, V. , & Pace, G. (2026). A review of guidelines for the diagnosis and treatment of borderline personality disorder and its features in adolescence. Personality and Mental Health, 20(2), e70069. 10.1002/pmh.70069 41846410

[aot70127-bib-0048] Ludowyke, L. , Lentin, P. , & Brown, T. (2024). The meaning and purpose of creativity in the daily life occupations, activities, acts and behaviors amongst adults living with mental health conditions: A scoping review. Occupational Therapy in Mental Health, 40(1), 45–85. 10.1080/0164212X.2023.2218121

[aot70127-bib-0049] McGowan, N. M. , & Saunders, K. E. (2021). The emerging circadian phenotype of borderline personality disorder: Mechanisms, opportunities and future directions. Current Psychiatry Reports, 23(5), 30. 10.1007/s11920-021-01236-w PMC8035096 33835306

[aot70127-bib-0050] Mohi, S. R. , Deane, F. P. , Bailey, A. , Mooney‐Reh, D. , & Ciaglia, D. (2018). An exploration of values among consumers seeking treatment for borderline personality disorder. Borderline Personality Disorder and Emotion Dysregulation, 5(1), 8. 10.1186/s40479-018-0085-9 PMC5899834 29686876

[aot70127-bib-0051] Nance, M. , Stetsiv, K. , McNamara, I. A. , Carpenter, R. W. , & Hepp, J. (2024). Acute, chronic, and everyday physical pain in borderline personality disorder. Current Psychiatry Reports, 26(5), 240–248. 10.1007/s11920-024-01498-0 PMC11082044 38598062

[aot70127-bib-0052] Petersen, J. , Palic, S. , Nyboe, L. , Schuster, M. , Videbech, P. , & Midtgaard, J. (2025). Physical activity in the management of borderline personality disorder: A scoping review. Psychiatry Research, 350, 116535. 10.1016/j.psychres.2025.116535 40398190

[aot70127-bib-0053] Portney, L. G. (2020). Foundations of clinical research: Applications to evidence‐based practice (4th ed.). F.A. Davis.

[aot70127-bib-0054] Sandelowski, M. (2000). Whatever happened to qualitative description? Research in Nursing & Health, 23(4), 334–340. 10.1002/1098-240X(200008)23:4<334::AID-NUR9>3.0.CO;2-G 10940958

[aot70127-bib-0055] Sandelowski, M. (2010). What's in a name? Qualitative description revisited. Research in Nursing & Health, 33(1), 77–84. 10.1002/nur.20362 20014004

[aot70127-bib-0056] Sansone, R. A. , & Wiederman, M. W. (2012). Spending too much: Relationships with borderline personality symptomatology. International Journal of Psychiatry in Clinical Practice, 16(4), 316–318. 10.3109/13651501.2012.693186 22978567

[aot70127-bib-0057] Stanley, M. (2023). Qualitative descriptive: A very good place to start. In Qualitative research methodologies for occupational science and occupational therapy (pp. 52–67). Routledge.

[aot70127-bib-0058] Steele, K. R. , Townsend, M. L. , & Grenyer, B. F. (2019). Parenting and personality disorder: An overview and meta‐synthesis of systematic reviews. PLoS ONE, 14(10), e0223038. 10.1371/journal.pone.0223038 PMC6772038 31574104

[aot70127-bib-0059] Steingrímsson, S. , Odéus, E. , Cederlund, M. , Franzén, S. , Helgesson, C. , Nyström, K. , Sondell, J. , & Opheim, A. (2022). Weighted blanket and sleep medication use among adults with psychiatric diagnosis–a population‐based register study. Nordic Journal of Psychiatry, 76(1), 29–36. 10.1080/08039488.2021.1931713 34106812

[aot70127-bib-0060] Stiles, C. , Batchelor, R. , Gumley, A. , & Gajwani, R. (2023). Experiences of stigma and discrimination in borderline personality disorder: A systematic review and qualitative meta‐synthesis. Journal of Personality Disorders, 37(2), 177–194. 10.1521/pedi.2023.37.2.177 37002935

[aot70127-bib-0061] Stoffers‐Winterling, J. M. , Ribeiro, J. P. , Kongerslev, M. T. , Völlm, B. A. , Mattivi, J. T. , Faltinsen, E. , Todorovac, A. , Jørgensen, M. S. , Callesen, H. E. , & Sales, C. P. (2022). Pharmacological interventions for people with borderline personality disorder. Cochrane Database of Systematic Reviews, 2022(11), CD012956. 10.1002/14651858.CD012956.pub2 PMC9662763 36375174

[aot70127-bib-0062] Stoffers‐Winterling, J. M. , Storebø, O. J. , Kongerslev, M. T. , Faltinsen, E. , Todorovac, A. , Jørgensen, M. S. , Sales, C. P. , Callesen, H. E. , Ribeiro, J. P. , & Völlm, B. A. (2022). Psychotherapies for borderline personality disorder: A focused systematic review and meta‐analysis. The British Journal of Psychiatry, 221(3), 538–552. 10.1192/bjp.2021.204 35088687

[aot70127-bib-0063] Storebø, O. J. , Stoffers‐Winterling, J. M. , Völlm, B. A. , Kongerslev, M. T. , Mattivi, J. T. , Kielsholm, M. L. , Nielsen, S. S. , Jørgensen, M. P. , Faltinsen, E. G. , Lieb, K. , & Simonsen, E. (2020). Psychological therapies for people with borderline personality disorder. Cochrane Database of Systematic Reviews, 2020(2), CD012955. 10.1002/14651858.CD012955 PMC7199382 32368793

[aot70127-bib-0064] Tepper, D. , Sellar, B. , Shipley, S. , Smith, R. , & Murray, C. M. (2025). A scoping review of programmes that address the daily living functioning of people diagnosed with borderline personality disorder. The Australian and New Zealand Journal of Psychiatry, 60, 747–764. 10.1177/00048674251393159 PMC13392200 41317141

[aot70127-bib-0065] Tong, A. , Sainsbury, P. , & Craig, J. (2007). Consolidated criteria for reporting qualitative research (COREQ): A 32‐item checklist for interviews and focus groups. International Journal for Quality in Health Care, 19(6), 349–357. 10.1093/intqhc/mzm042 17872937

[aot70127-bib-0066] Trainor, L. R. , & Bundon, A. (2021). Developing the craft: Reflexive accounts of doing reflexive thematic analysis. Qualitative Research in Sport, Exercise and Health, 13(5), 705–726. 10.1080/2159676X.2020.1840423

[aot70127-bib-0067] van Trigt, S. , van der Zweerde, T. , van Someren, E. J. , van Straten, A. , & van Marle, H. J. (2025). A theoretical perspective on the role of sleep in borderline personality disorder: From causative factor to treatment target. Sleep Medicine Reviews, 81, 102089. 10.1016/j.smrv.2025.102089 40258322

[aot70127-bib-0068] Weideman, Z. (2025). Assessment and treatment of personality disorders in occupational therapy practice. In T. Machingura (Ed.), Occupational therapy practice in mental health: Models, conditions, interventions, and recovery (pp. 119–137). Bentham Science Publishers. 10.2174/97898153137101250101

[aot70127-bib-0069] Winsper, C. , Tang, N. K. , Marwaha, S. , Lereya, S. T. , Gibbs, M. , Thompson, A. , & Singh, S. P. (2017). The sleep phenotype of borderline personality disorder: A systematic review and meta‐analysis. Neuroscience & Biobehavioral Reviews, 73, 48–67. 10.1016/j.neubiorev.2016.12.008 27988314

[aot70127-bib-0070] Wood, A. , Brooks, R. , & Beynon‐Pindar, C. (2015). The experience of sleep for women with borderline personality disorder: An occupational perspective. British Journal of Occupational Therapy, 78(12), 750–756. 10.1177/0308022615587864

[aot70127-bib-0071] Woodbridge, J. , Townsend, M. , Reis, S. , Singh, S. , & Grenyer, B. F. (2022). Non‐response to psychotherapy for borderline personality disorder: A systematic review. The Australian and New Zealand Journal of Psychiatry, 56(7), 771–787. 10.1177/00048674211046893 PMC9218414 34525867

[aot70127-bib-0072] World Health Organization . (2022). ICD‐11: International classification of diseases (11th revision). https://icd.who.int/en/

[aot70127-bib-0073] Yeomans, F. E. , Clarkin, J. F. , & Kernberg, O. F. (2015). Transference‐focused psychotherapy for borderline personality disorder: A clinical guide. American Psychiatric Publishing.

[aot70127-bib-0074] Young, J. E. , Klosko, J. S. , & Weishaar, M. E. (2003). Schema therapy: A practitioner's guide. Guilford Press.

[aot70127-bib-0075] Zanarini, M. C. , Frankenburg, F. R. , Reich, D. B. , & Fitzmaurice, G. (2010). The 10‐year course of psychosocial functioning among patients with borderline personality disorder and axis II comparison subjects. Acta Psychiatrica Scandinavica, 122(2), 103–109. 10.1111/j.1600-0447.2010.01543.x PMC3876887 20199493

